# Clinical and genetic spectrum of inborn errors of immunity: a retrospective study on outcomes at a single center

**DOI:** 10.3389/fimmu.2026.1758410

**Published:** 2026-02-02

**Authors:** Hulya Kose, Akcahan Akalin

**Affiliations:** 1 Department of Pediatric Immunology and Allergy, Diyarbakir Children’s Hospital, Diyarbakir, Türkiye; 2 Department of Pediatric Genetics, Diyarbakir Children’s Hospital, Diyarbakir, Türkiye

**Keywords:** ADA deficiency, gene, inborn errors of immunity, IVIG, LIG4

## Abstract

**Introduction:**

Inborn errors of immunity (IEI) are particularly prevalent in regions with high rates of consanguinity, yet the genetic profiles in these populations are underreported. This study aims to describe the clinical and molecular characteristics of IEI in a highly consanguineous population and investigate the impact of genetic diagnosis on patient management.

**Method:**

This retrospective study included 52 patients with suspected IEI, as defined by the IUIS criteria. Clinical, immunological, and demographic data were recorded. Genetic analyses were performed primarily using next-generation sequencing (NGS) gene panels, and all pathogenic variants were confirmed by Sanger sequencing. Variants were interpreted in accordance with the ACMG guidelines.

**Results:**

A total of 52 patients were included in the study, with 92% of the individuals born to consanguineous parents, comprising 28 females and 24 males. The mean age at diagnosis was 4.63 ± 2.5 years. The median duration of follow-up was three years. The overall incidence was 0.3% representing the proportion of patients diagnosed with IEI among those referred to our center during the study period. A high rate of consanguineous marriage was observed, reported in 92% of the cases. The most frequently represented category was Predominantly Antibody Deficiencies (PAD), accounting for 20 patients (38.5%), including 12 cases (23%) of transient hypogammaglobulinemia of infancy (THI) and 7 cases (13%) of selective IgA deficiency. Among the 52 patients, 3 (5.8%) were diagnosed with severe combined immunodeficiency (SCID): 1 patient had ADA deficiency, and two patients had DNA ligase IV deficiency (LIG4). Additionally, 14 patients (26%) were diagnosed with combined Immunodeficiencies (CID). Thirty patients were treated with IVIG, and 3 patients underwent HSCT. A molecular diagnosis was established in 33 patients (63%). Genetic findings influenced clinical management in 82% of variant-positive cases, including decisions regarding HSCT, targeted therapy, and genetic counseling.

**Conclusion:**

This study highlights the distinctive genetic characteristics of IEI in a population with high consanguinity, emphasizing the need to incorporate molecular diagnostics into standard immunology practice, particularly in areas where recessive disorders are prevalent.

## Introduction

Inborn errors of immunity are inherited conditions that impair either the innate or adaptive immune systems. These disorders increase susceptibility to infections and may lead to immune dysregulation. The estimated incidence of IEIs ranges from 1 in 1,000 to 1 in 2,000 live births, with significant morbidity and mortality rates (1). Advances in molecular diagnostic tools have identified approximately 500 genetic defects associated with these conditions, and this number is expected to increase (2–4).

Epidemiological data on IEIs vary widely by region, influenced by factors such as genetic background, testing availability, and clinical awareness. Populations with high consanguinity rates tend to have a higher prevalence of autosomal recessive IEIs, though detailed studies on disease patterns in these groups are limited. While the understanding of IEIs now extends from infectious susceptibility to include immune dysregulation conditions, such as autoimmunity, inflammation, and cancer, many studies have yet to fully characterize these features (5, 6). Molecular diagnosis is crucial; however, data on diagnostic delays, barriers to genetic testing, and the impact of early diagnosis on outcomes remain limited, especially in low-resource settings. Despite the high rate of consanguineous marriages in our region, making such cases less common, we highlight the most significant ones.

Our region in southeastern Türkiye has a high prevalence of consanguineous marriages, likely increasing the occurrence of autosomal recessive and syndromic IEIs. This study aimed to characterize the clinical and genetic features of IEI patients at a single secondary pediatric center in an area with high consanguinity. We also evaluated how molecular diagnosis influenced their management. By sharing data on IEI category distribution and presenting rare cases, we aim to provide region-specific insights that can enhance diagnostic suspicion, inform genetic testing strategies, and support genetic counseling in similar settings.

## Methods

### Patient recruitment

We included all consecutive patients referred to our immunology department between 2022–2025 with suspected or confirmed IEI and with at least 12 months of follow-up. Ig levels were measured by nephelometry, and lymphocyte subgroups were analyzed by flow cytometry. We obtained ethics approval number 198 on February 10, 2024, from Gazi Yasargil Training Hospital. The study was conducted in accordance with the principles outlined in the Declaration of Helsinki. Written informed consent was taken from the parents.

### Molecular analysis

Next-generation sequencing (NGS) was used to analyze the exome, covering approximately 60 megabases of the human genome. Target enrichment was achieved with the Roche Kapa HyperExome kit, ensuring 90% coverage. Sequencing was performed on the MGI DNBSEQ-G400 platform, yielding an average read depth of 20X and 99.25% coverage. Bioinformatic analysis was conducted using Genomize Seq software (version 6.6.0), which was aligned to the human reference genome (GRCh37/hg19). Low-coverage regions and likely artifact variants were excluded. Variants were annotated using ClinVar, HGMD, and ExAC. All identified variants were classified for pathogenicity according to the ACMG guidelines (7). Whole-exome sequencing performed in selected cases with inconclusive panel results or atypical phenotypes.

### Patient selection and inclusion criteria

1. All consecutive patients were eligible if they met these criteria:
2. Clinical suspicion of IEI based on recurrent, severe, persistent, or unusual infections; immune dysregulation; or syndromic features.
3. Availability of immunological evaluation, including serum immunoglobulin levels and lymphocyte subset analysis.
4. Completion of genetic analysis through next-generation sequencing (NGS) or targeted testing.
5. Follow-up duration of at least 12 months.

### Clinical and immunological evaluation

Demographic information, including age, sex, parental consanguinity, family history, age at symptom onset, and age at diagnosis, was gathered from medical records. Serum immunoglobulin levels (IgG, IgA, IgM, IgE) were determined using nephelometry (Beckman Coulter AU5800). Lymphocyte subsets (CD3+, CD4+, CD8+, CD19+, and NK cells) were analyzed through 6-color flow cytometry (BD FACSCanto II). Patients were classified according to the 2024 guidelines of the International Union of Immunological Societies (IUIS) (8).

### Treatment and follow-up

Recorded management strategies included immunoglobulin replacement, antimicrobial prophylaxis, enzyme replacement therapy, and hematopoietic stem cell transplantation (HSCT).

## Results

A total of 52 patients with suspected or confirmed inborn errors of immunity were evaluated. The cohort included 24 males and 28 females, with a mean age at diagnosis of 4.63 ± 2.5 years. Parental consanguinity was present in 92% of the cohort. The median follow-up duration was 3 years.

### Distribution of IEI categories

**Table 1** displays the distribution of immunological diagnoses across IUIS categories. The largest group was primarily antibody deficiencies (n = 20, 38.5%), which included 12 cases (23%) of transient hypogammaglobulinemia of infancy (THI) and 7 cases (13%) of selective IgA deficiency. Combined immunodeficiencies with or without syndromic features were observed in 14 patients (26%). These included:

**Table 1:** The distribution of the cohort.

IUIS category	Diagnosis	Gene	cDNA variant	Protein change	Zygosity	n	ACMG classification*	ACMG criteria Used	In silico prediction
SCID	ADA deficiency	*ADA*	c.956_960del; c.845G>A	p.E319Gfs*3; p.R282Q	Compound	1	Pathogenic	PVS1, PM2_supporting, PP4	LOF; missense predicted damaging
SCID	LIG4 deficiency	*LIG4*	c.73C>T	p.R25*	Homozygous	2	Pathogenic	PVS1, PM2_supporting, PP4	LOF
CID	IP	*IKBKG*	c.1167dupC	p.E390Rfs*5	Hemizygous	1	Pathogenic	PVS1, PM2_supporting, PP4	LOF
CID	SIOD	*SMARCAL1*	c.1027_1034del; c.2459G>A	p.F343Rfs*13; p.R820H	Homozygous	2	Pathogenic	PVS1, PM3_supporting, PP4	LOF + missense damaging
CID	AT	*ATM*	c.4940T>G	p.L1647R	Homozygous	2	Likely pathogenic	PM2_supporting, PP3, PP4	Damaging
CID	AT	*ATM*	c.1339C>T	p.R447*	Homozygous	2	Pathogenic	PVS1, PM2_supporting, PP4	LOF
CID	DGS	22q11.21del	—	—	—	1	Pathogenic	Well-established pathogenic del	—
CID	Bloom	*BLM*	c.2014C>T; c.2074 + 2T>C	p.Q672*; splice	Homozygous	2	Pathogenic	PVS1, PM2_supporting, PP4	LOF
CID	HIES	*IL6ST*	c.2155dup	p.I719Nfs*2	Heterozygous	1	Pathogenic	PVS1, PM2_supporting, PP4	LOF
CID	HIES	*IL6R*	c.923C>T	p.P308L	Homozygous	1	Likely pathogenic	PM2_supporting, PP3, PP4	Damaging
CID	Loeys–Dietz	*TGFBR1*	c.1025A>G	p.K342R	Heterozygous	1	Likely pathogenic	PM1, PM2_supporting, PP3, PP4	Damaging
CID	RTS	*RECQL4*	c.2462dup	p.E823Rfs*61	Homozygous	1	Pathogenic	PVS1, PM2_supporting, PP4	LOF
PAD	TACI deficiency	*TNFRSF13B*	c.515G>A	p.C172Y	Heterozygous	1	Likely pathogenic	PM1, PM2_supporting, PP3	Damaging
PAD	Selective IgA deficiency	—	—	—	—	7	NA	—	—
PAD	THI	—	—	—	—	12	NA	—	—
Thymic defects	FOXN1 def.	*FOXN1*	c.56T>C	p.L19P	Heterozygous	1	VUS/Likely pathogenic	PM2_supporting, PP3	Damaging
Immune dysregulation	IPEX	*FOXP3*	c.1117_1118delinsGC	p.F373A	Hemizygous	1	Pathogenic	PVS1, PM2_supporting, PP4	LOF
Immune dysregulation	NF-κB defect	*RIPK1*	c.1169G>T	p.R390L	Homozygous	1	Likely pathogenic	PM2_supporting, PP3, PP4	Damaging
Congenital neutropenia	Cohen	*VPS13B*	c.412 + 1G>T; c.3529G>T	splice; p.E1177*	Homozygous	2	Pathogenic	PVS1, PM3_supporting, PP4	LOF
Congenital neutropenia	G6PC3 def.	*G6PC3*	c.175T>C	p.W59R	Homozygous	1	Likely pathogenic	PM2_supporting, PP3, PP4	Damaging
Congenital neutropenia	GFI1 def.	*GFI1*	c.481C>T	p.P16S	Heterozygous	1	VUS/Likely pathogenic	PM2_supporting, PP3	Damaging
MSMD	STAT1 def.	*STAT1*	c.1341C>A	p.D447E	Heterozygous	1	VUS	PM2_supporting, PP3	Possibly damaging
IFNopathies	SPENCDI	*ACP5*	c.772_790del	p.S258Wfs*39	Homozygous	1	Pathogenic	PVS1, PM2_supporting, PP4	LOF
Non-hematopoietic	Osteopetrosis	*CLCN7*	c.1577G>A	p.R526Q	Homozygous	1	Likely pathogenic	PM2_supporting, PP3, PP4	Damaging
Inflammasome	PLAID	*PLCG2*	c.702C>G	p.D234E	Heterozygous	1	VUS/Likely pathogenic	PM2_supporting, PP3	Damaging
Low Ig	ATP6AP1 def.	*ATP6AP1*	c.673C>T	p.R225C	Hemizygous	1	Likely pathogenic	PM2_supporting, PP3, PP4	Damaging
CMC	IL-17RC def.	*IL17RC*	c.991C>T	p.R331W	Homozygous	1	Likely pathogenic	PM2_supporting, PP3, PP4	Damaging
Bone marrow failure	FA	*FANCE*	c.355C>T	p.Q119*	Homozygous	1	Pathogenic	PVS1, PM2_supporting, PP4	LOF

SCID, Severe combined immunodeficiency; CID, Combined immunodeficiency; ADA, Adenosine deaminase deficiency; IP, Incontinentia pigmenti; AT, Ataxia-Telangiectasia; DGS, DiGeorge Syndrome; HIES, Hyper-IgE Syndrome; Aplaid, Autoinflammation and antibody deficiency; SIOD, Schimke Immuno-osseous dysplasia; IPEX, Immune dysregulation, polyendocrinopathy, enteropathy X-linked; SPENCD, Spondylochondrodysplasia with immune dysregulation; THI, Transient hypogammaglobulinemia of infancy; FA, Fanconi anemia.

* means the stop codon

- *ADA* deficiency (1 patient; compound heterozygous *c.956_960del* and *c.845G>A*)
- *LIG4* deficiency (2 patients; homozygous *c.73C>T*)
- Schimke immuno-osseous dysplasia due to *SMARCAL1* variants (2 patients)
- Incontinentia pigmenti due to *IKBKG* frameshift mutation (1 patient)
- Ataxia-telangiectasia due to *ATM* variants (4 patients)
- Bloom syndrome (*BLM* variants) (2 patients)
- DiGeorge syndrome (1 patient), and other combined defects as listed in **Table 1**.

### Syndromic IEIs and immune dysregulation disorders

One patient was found to have a heterozygous loss-of-function mutation in FOXP3, indicating IPEX syndrome. Two other patients exhibited ectodermal dysplasia combined with immunodeficiency: one with RIPK1 deficiency and the other with a pathogenic IKBKG variant. Additionally, one patient had a homozygous IL6R mutation linked to a hyper-IgE phenotype, while another had a heterozygous frameshift mutation in IL6ST. Finally, one patient was diagnosed with a PLCG2-related autoinflammatory condition with antibody deficiency, known as APLAID.

### Congenital neutropenias and bone marrow failure

- Four patients were diagnosed with congenital neutropenia, including:
- Two with Cohen syndrome due to biallelic VPS13B variants
- One with G6PC3-related neutropenia
- One with GFI1 deficiency

Bone marrow failure due to *the FANCE pathogenic variant was diagnosed in one patient, while congenital osteopetrosis due to the CLCN7* mutation was identified in another.

### Other rare IEIs

One patient exhibited type I interferonopathy (SPENCDI) due to a homozygous variant in ACP5. Another patient was diagnosed with Rothmund–Thomson syndrome, which involves a RECQL4 frameshift mutation. Additionally, an infant presenting with liver dysfunction was found to have a congenital disorder of glycosylation related to ATP6AP1 (**Table 1**).

### Genetic confirmation

A molecular diagnosis was confirmed in 33 of 52 patients (63.5%). In total, 37 pathogenic or likely pathogenic variants were identified across 23 genes. All pathogenic variants were validated using Sanger sequencing. Details on variant pathogenicity, ACMG classification, allele frequency (gnomAD), and in silico predictions (SIFT, PolyPhen-2) are provided in **Table 2**.

**Table 2:** Clinical, genetic, and phenotypic findings of the rare cases.

Case	Age/sex	Key clinical features	Genetic variant (HGVS)	Final diagnosis (IUIS category)	Treatment & outcome	Figures
1	8.5 y/M	Severe growth delay, microcephaly, proteinuria, skeletal anomalies, cerebral atrophy	*SMARCAL1* (NM_014140.4) c.1027_1034del, p.(Phe343Argfs*13), homozygous	SIOD	ACE inhibitor + SCIG; chronic kidney disease under follow-up	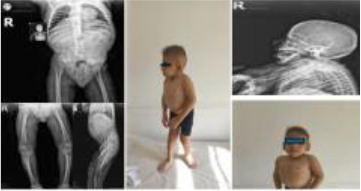
2	1 y/F	Microcephaly, growth failure, hypogammaglobulinemia	*LIG4* (NM_206937.2) c.73C>T, p.(Arg25*), homozygous	LIG4 deficiency	HSCT performed; improved post-transplant	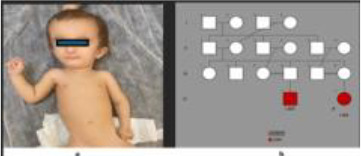
3	1.5 y/M	Microcephaly, hypogonadism, growth failure, hypogammaglobulinemia	*LIG4* (same as above), homozygous	LIG4 deficiency	IVIG; family declined HSCT	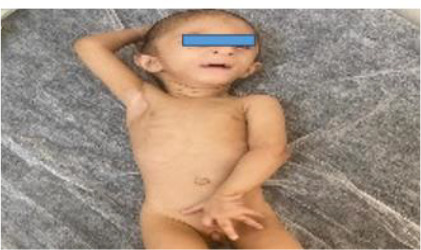
4	Infant/M	SCID phenotype, severe lymphopenia, lung infection	*ADA* (NM_000022.4) c.956_960del, p.(Glu319Glyfs*3) & c.845G>A, p.(Arg282Gln), compound heterozygous	ADA-SCID	Enzyme replacement + HSCT; full donor chimerism	–
5	Child/F	Butterfly-type malar rash, growth delay, hyperpigmentation, hypogammaglobulinemia	*BLM* (NM_000057.4) c.2014C>T, p.(Gln672*) homozygous	Bloom Syndrome	IVIG; dermatologic follow-up	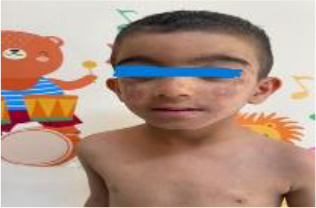
6	4 y/M	Recurrent fevers, sweating defect, alopecia, oligodontia, mild cognitive delay	*RIPK1* (NM_001354930.2) c.1169G>T, p.(Arg390Leu), homozygous	RIPK1-associated immune dysregulation	Colchicine → inflammatory markers decreased	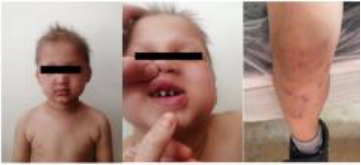
7	7 y/F	Recurrent lung infections, diarrhea, epilepsy, sparse hair, conical teeth, hyperpigmentation (23).	*IKBKG* (NM_001099857.5) c.1167dup, p.(Glu390Argfs*5) heterozygous	Incontinentia Pigmenti with immunodeficiency	IVIG q3 weeks + azathioprine; stable	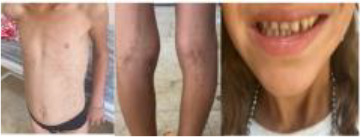
8	1.5 mo/M	Infantile liver dysfunction, elevated transaminases; family history of ADA-SCID	*ATP6AP1* (NM_001183.6) c.673C>T, p.(Arg225Cys) hemizygous	Congenital disorder of glycosylation	Supportive; liver enzymes normalized by 5 months	–
9	10.5 y/M	Neutropenia, oral aphthae, dysmorphic features, hyperflexibility, truncal obesity	*VPS13B* (NM_152564.5) c.3529G>T, p.(Glu1177*) homozygous	Cohen Syndrome	Supportive; multidisciplinary follow-up	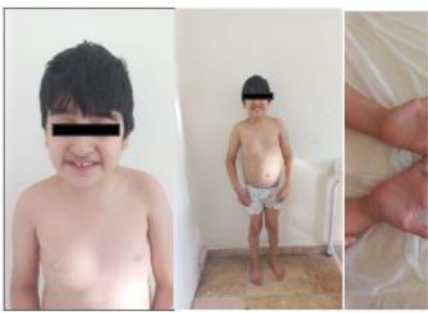
10	19 mo/F	Bilateral absent radius, hearing loss, corpus callosum agenesis, microcephaly, growth delay	*RECQL4* (NM_004260.4) c.2462dup, p.(Glu823Argfs*61), homozygous	Rothmund–Thomson Syndrome	Multisystemic management; high malignancy risk	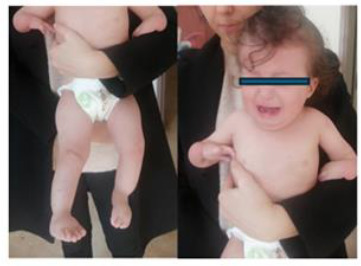

### Impact on clinical management

After molecular confirmation, 30 patients (57.6%) received immunoglobulin replacement therapy (either IVIG or SCIG). Three patients (5.8%) underwent hematopoietic stem cell transplantation (HSCT), including cases with LIG4 deficiency and ADA-SCID. Patients with immune dysregulation (such as FOXP3, RIPK1, and IKBKG mutations) require targeted immunomodulatory treatments. Additionally, one patient with ADA deficiency was treated with enzyme replacement therapy before undergoing HSCT.

### Immunoglobulin profiles and indications for IgG replacement therapy

Immunoglobulin replacement therapy was administered to 30 of 52 patients (57.6%). The majority of patients receiving IgG replacement were diagnosed with combined immunodeficiencies, DNA-repair disorders, immune dysregulation syndromes, or predominantly antibody deficiencies. In contrast, only a subset of patients with transient hypogammaglobulinemia of infancy required short-term IgG replacement therapy due to recurrent or severe infections. Baseline immunoglobulin levels, antibody responses, and indications for IgG replacement therapy are summarized in **Table 3**.

**Table 3:** Immunoglobulin profiles, antibody responses, and IgG replacement therapy status.

Diagnosis	n	Pre-treatment IgG (median, mg/dL)	IgA (median, mg/dL)	IgM (median, mg/dL)	Vaccine antibody response	IVIG required	Clinical course/outcome
SCID/CID (ADA, LIG4)	4	278 (276–371)	0.05 (0.05–0.05)	41 (32–233)	Impaired	Yes (4/4)	HSCT or long-term IVIG
DNA-repair disorders (ATM)	4	394 (357–431)	0.06 (0.05–0.08)	261 (118–413)	Impaired (4/4)	Yes (3/4)	Chronic follow-up
Immune dysregulation (IPEX, IKBKG, NF-κB pathway)	5	402 (242–467)	15 (0.59–38)	37 (11–58)	Impaired (3/5)	Yes (4/5)	Stable under treatment
Predominantly antibody deficiency (non-THI)	7	280 (224–401)	0.05 (0.01–49)	40 (29–87)	Impaired (5/7)	Yes (6/7)	Ongoing IVIG
Transient hypogammaglobulinemia of infancy (THI)	12	227 (171–305)	0.08 (0.01–15)	40 (29–87)	Preserved (9/12)	Yes (4/12)	IgG normalized at 24–36 months

## Discussion

This single-center cohort from a region with high consanguinity rates revealed a wide range of inborn errors of immunity, predominantly involving autosomal recessive and syndromic variants. Over 90% of patients had consanguineous parents, a figure higher than that reported in many regional studies from the Middle East, North Africa, and South Asia, where consanguinity usually ranges from 40% to 70% (9–11). This demographic factor likely explains the high incidence of rare disorders in our cohort, such as those associated with LIG4, SMARCAL1, BLM, RECQL4, RIPK1, and ATP6AP1. Most of these rare cases are detailed in **Table 2**. Similar patterns of increased DNA-repair disorders, interferonopathies, and congenital neutropenias have been observed in other consanguineous populations, supporting the genetic epidemiology consistent with our region.

Antibody deficiencies constituted the largest clinical group, consistent with previous international and regional reports (12–14). The significant number of transient hypogammaglobulinemia cases of infancy also highlights our center’s role as the primary pediatric allergy and immunology clinic serving a large catchment area. Molecular diagnosis was successful in 63.5% of the cohort. Notably, genetic confirmation directly influenced treatment decisions in over half of the patients, leading to interventions such as immunoglobulin replacement therapy, targeted immunomodulation, enzyme replacement, or hematopoietic stem cell transplantation. These results underscore the importance of early access to genomic testing, especially in regions where autosomal recessive IEIs are common and clinical signs may overlap or be atypical. The detection of variants across 23 genes emphasizes the diversity of IEIs in populations with high genetic load. Several variants in our cohort were rare or had limited representation in public databases, highlighting the need for region-specific genomic data. The ACMG classification, in silico predictions, and available functional literature supported the pathogenicity (5). Although functional assays were not conducted in this study, many of the variants identified—such as those in ADA, LIG4, FOXP3, IKBKG, and PLCG2—are known to have well-established disease mechanisms, as reported in previous studies (15–22).

Overall, our findings suggest that combining clinical assessments, immunological tests, and genetic sequencing improves diagnostic precision and facilitates the development of personalized treatment plans. The frequent appearance of severe conditions like SCID, DNA-repair disorders, autoinflammatory syndromes, and congenital neutropenia highlights the importance of genetic counseling in communities with high consanguinity. In such groups, characterized by increased consanguinity, we identified a wide range of inherited immune disorders, including numerous rare autosomal recessive and syndromic cases. Early molecular diagnosis has significantly impacted patient management, enabling targeted treatments such as immunoglobulin replacement, immunomodulation, and stem cell transplantation. Our results underscore the crucial need for accessible genetic testing, enhanced clinician awareness, and comprehensive genetic counseling in regions with high rates of consanguinity.

Our cohort showed that patients with combined immunodeficiencies, DNA-repair disorders, and immune dysregulation syndromes consistently exhibit low IgG levels and defective antibody production, highlighting the need for ongoing immunoglobulin replacement therapy. Conversely, most patients with transient hypogammaglobulinemia maintained adequate vaccine responses and experienced spontaneous normalization of their immunoglobulin levels, allowing for the safe discontinuation of long-term treatment. These results underscore the importance of continuous immunological monitoring and integrating genetic and functional assessments to prevent unnecessary treatments and ensure timely care for patients with permanent immunodeficiency.

This study has some limitations. Firstly, it was carried out at only one center and may not reflect all IEI cases in the area, especially milder cases that may not have been referred. Secondly, functional validation of new or rare variants was not done; instead, pathogenicity was assessed using ACMG criteria, database annotations, segregation analysis, and existing literature.

## Conclusion

These cases demonstrate how combining clinical suspicion with genetic testing facilitates the early identification of rare immunodeficiency disorders. Early diagnosis allows for treatments like IVIG, prophylactic therapies, or potentially curative HSCT. Many related syndromes also pose cancer and systemic risks, requiring a multidisciplinary approach and ongoing monitoring.

## Data Availability

The original contributions presented in the study are included in the article/supplementary material. Further inquiries can be directed to the corresponding author.

## References

[1] Bonilla FA Khan DA Ballas ZK Chinen J Frank MM Hsu JT . Practice parameters for the diagnosis and management of primary immunodeficiency. J Allergy Clin Immunol. (2015) 136:1186–205.e1-78. doi: 10.1016/j.jaci.2015.04.049, PMID: 26371839

[2] Yu JE . New primary immunodeficiencies 2023 update. Curr Opin Pediatr. (2024) 36:112–23. doi: 10.1097/MOP.0000000000001315, PMID: 38001560

[3] Picard C Bobby Gaspar H Al-Herz W Bousfiha A Casanova JL Chatila T . International Union of Immunological Societies: 2017 Primary Immunodeficiency Diseases Committee Report on inborn errors of immunity. J Clin Immunol. (2018) 38:96 128. doi: 10.1007/s10875-017-0464-9, PMID: 29226302 PMC5742601

[4] Buckley RH Orange JS . Primary immunodeficiency diseases. In: Adkinson NF Bochner BS Burks AW Busse WW Holgate ST Lemanske RF , editors. Middleton’s allergy principles and practice, 8th ed. Saunders, Philadelphia (2014). p. 1144–1174. doi: 10.23822/EurAnnACI.1764-1489.239, PMID:

[5] Rasouli SE Tavakol M Sadri H Chavoshzadeh Z Alireza Mahdaviani S Delavari S . The spectrum of inborn errors of immunity: a single tertiary center retrospective study in Alborz, Iran. Eur Ann Allergy Clin Immunol. (2023) 55:19–28. doi: 10.23822/EurAnnACI.1764-1489.239, PMID: 34918886

[6] Al Farsi T Ahmed K Alshekaili J Al Kindi M Cook M Al-Hosni A . Immune dysregulation in monogenic inborn errors of immunity in Oman: over A decade of experience from a single tertiary center. Front Immunol. (2022) 13:849694. doi: 10.3389/fimmu.2022.849694, PMID: 35464432 PMC9019296

[7] Richards S Aziz N Bale S Bick D Das S Gastier-Foster J . Standards and guidelines for interpreting sequence variants: a joint consensus recommendation of the American College of Medical Genetics and Genomics and the Association for Molecular Pathology. Genet Med. (2015) 17:405–24. doi: 10.1038/gim.2015.30, PMID: 25741868 PMC4544753

[8] Tangye SG Al-Herz W Bousfiha A Cunningham-Rundles C Franco JL Holland SM . Human inborn errors of immunity: 2024 update on the classification from the International Union of Immunological Societies Expert Committee. J Clin Immunol. (2022) 42:1473–1507. doi: 10.1007/s10875-022-01289-3, PMID: 35748970 PMC9244088

[9] Belaid B Lamara Mahammed L Drali O Oussaid AM Touri NS Melzi S . Inborn errors of immunity in Algerian children and adults: A single-center experience over a period of 13 years (2008-2021). Front Immunol. (2022) 13:900091. doi: 10.3389/fimmu.2022.900091, PMID: 35529857 PMC9069527

[10] Al Sukaiti N Ahmed K Alshekaili J Al Kindi M Cook MC Farsi TA . A decade experience on severe combined immunodeficiency phenotype in Oman, bridging to newborn screening. Front Immunol. (2021) 11:623199. doi: 10.3389/fimmu.2020.623199, PMID: 33519828 PMC7844122

[11] Firatoglu H Aytekin C Dogu F Bal SK Haskologlu S Boztug K . Evaluation of patients with combined immunodeficiency: A single center experience. Iran J Immunol. (2025) 22:89–99. doi: 10.22034/iji.2025.103499.2844, PMID: 40040385

[12] Kapousouzi A Kalala F Sarrou S Farmaki E Antonakos N Kakkas I . Nationwide study of the delayed diagnosis and the clinical manifestations of predominantly antibody deficiencies and *CTLA4*-mediated immune dysregulation syndrome in Greece. Med (Kaunas). (2024) 60:782. doi: 10.3390/medicina60050782, PMID: 38792965 PMC11123397

[13] Azizi G Bagheri Y Tavakol M Askarimoghaddam F Porrostami K Rafiemanesh H . The clinical and immunological features of patients with primary antibody deficiencies. Endocr Metab Immune Disord Drug Targets. (2018) 18:537–45. doi: 10.2174/1871530318666180413110216, PMID: 29651973

[14] Wang LJ Yang YH Lin YT Chiang BL . Immunological and clinical features of pediatric patients with primary hypogammaglobulinemia in Taiwan. Asian Pac J Allergy Immunol. (2004) 22:25–31., PMID: 15366655

[15] Saraiva JM Dinis A Resende C Faria E Gomes C Correia AJ . Schimke immuno-osseous dysplasia: case report and review of 25 patients. J Med Genet. (1999) 36:786–9. doi: 10.1136/jmg.36.10.786, PMID: 10528861 PMC1734237

[16] Schober S Schilbach K Doering M Cabanillas Stanchi KM Holzer U Kasteleiner P . Correction to: Allogeneic hematopoietic stem cell transplantation in two brothers with DNA ligase IV deficiency: a case report and literature review. BMC Pediatr. (2019) 19:470. doi: 10.1186/s12887-019-1851-6, PMID: 31791281 PMC6886177

[17] German J Sanz MM Ciocci S Ye TZ Ellis NA . Syndrome-causing mutations of the BLM gene in persons in the Bloom’s Syndrome Registry. Hum Mutat. (2007) 28:743–53. doi: 10.1002/humu.20501, PMID: 17407155

[18] Larizza L Roversi G Volpi L . Rothmund-thomson syndrome. Orphanet J Rare Dis. (2010) 5:2. doi: 10.1186/1750-1172-5-2, PMID: 20113479 PMC2826297

[19] Zhou Q Lee GS Brady J Datta S Katan M Sheikh A . A hypermorphic missense mutation in PLCG2, encoding phospholipase Cγ2, causes a dominantly inherited autoinflammatory disease with immunodeficiency. Am J Hum Genet. (2012) 91:713–20. doi: 10.1016/j.ajhg.2012.08.006, PMID: 23000145 PMC3484656

[20] Santisteban I Arredondo-Vega FX Bali P Dalgic B Lee HH Kim M . Evolving spectrum of adenosine deaminase (ADA) deficiency: Assessing genotype pathogenicity according to expressed ADA activity of 46 variants. J Allergy Clin Immunol. (2025) 155:166–75. doi: 10.1016/j.jaci.2024.08.014, PMID: 39182630

[21] Narayanan MJ Rangasamy S Narayanan V . Incontinentia pigmenti (Bloch-Sulzberger syndrome). Handb Clin Neurol. (2015) 132:271–80. doi: 10.1016/B978-0-444-63432-0.00014-5 26564087

[22] Raynor A Lebredonchel É Foulquier F Fenaille F Bruneel A . Diagnostic and therapeutic approaches in congenital disorders of glycosylation. Handb Exp Pharmacol. (2025) 288:211–241. doi: 10.1007/164_2023_901, PMID: 40119203

[23] Köse H Akalın A . Ektodermal displazili hastaların klinik ve immunolojik özellikleri. Dicle Med J. (2025) 52:135–43. doi: 10.5798/dicletip.1582011

